# Susceptibility of MMP3 gene polymorphism to coronary artery disease: A meta-analysis

**DOI:** 10.5937/jomb0-43315

**Published:** 2023-10-27

**Authors:** Liu Wenwang

**Affiliations:** 1 Beijing Hospital of Integrated Chinese & Western Medicine, Department of Clinical Laboratory, Beijing China

**Keywords:** MMP3, CAD, polymorphism, MMP3, CAD, polimorfizam

## Abstract

**Background:**

Conclusions on susceptibility of MMP3-1612 5A/6A to morbid risk of coronary artery disease (CAD) are controversial. This meta-analysis aims to obtain the accurate relationship between them.

**Methods:**

Relevant literatures on susceptibility of MMP3-1612 5A/6A to morbid risk of CAD published before July 2019 were searched in PubMed, Web of Science, Cochrane Library, CNKI, VIP and Wanfang. Data were extracted from eligible literatures and analyzed by RevMan5.3 and STATA12.0 for calculating OR and corresponding 95% CI. Study selection: A total of 18 literatures reporting MMP3-1612 5A/6A and CAD were enrolled. Data extraction was conducted by two researches independently. Any disagreement was solved by the third research.

## Abstract (sr)

**Uvod:**

Zaključci o podložnosti MMP3 -1612 5A/6A morbidnom riziku od koronarne arterijske bolesti (CAD) su kontroverzni. Ova meta-analiza ima za cilj da dobije tačan odnos između njih.

**Metode:**

Relevantna literatura o podložnosti MMP3 -1612 5A/6A morbidnom riziku od CAD objavljena pre jula 2019. pretražena je u PubMed-u, Web of Science, Cochrane Library, CNKI, VIP i Wanfang. Podaci su izvučeni iz kvalifikovane literature i analizirani od strane RevMan5.3 i STATA12.0 za izračunavanje OR i odgovaraju}i 95% CI. Izbor studije: Ukupno 18 literatura koja izveštava o MMP3-1612 5A/6A i CAD je upisano. Ekstrakciju podataka sprovela su dva istraživanja nezavisno. Bilo kakvo neslaganje je rešeno trećim istraživanjem.

**Rezultati:**

U recesivnom modelu (OR=1,3, 95% CI=1,2-1,64, P=0,03) i naddominantnom modelu (OR=1,50, 95% CI=1,14-1,97, P=0,002), MMP3 -1612 5A/6A je u korelaciji sa osetljivo{}u na CAD. Analiza podgrupe je otkrila da je u različitim genetskim modelima, MMP3 -1612 5A/6A bio u korelaciji sa osetljivo{}u na CAD u populaciji istočne Azije (uglavnom u kineskoj populaciji).

**Zaključak:**

MMP3 -1612 5A/6A je usko povezan sa morbidnim rizikom od CAD. Interakcija GEN-sredina je fokus istraživanja u budu}im analizama.

## Introduction

Coronary artery disease (CAD) is a heart disease caused by stenosis or obstruction of the coronary artery lumen due to coronary atherosclerosis, as well as functional changes that lead to myocardial ischemia, hypoxia, or necrosis. CAD is also referred to as ischemic heart disease, and it is the most common subtype of atherosclerosis-related lesions that significantly impact human health [1]. CAD primarily affects individuals over 40 years of age, and it is more prevalent in men than in women. In recent years, the incidence of CAD has markedly increased in our country [1].

The matrix metalloproteinase (MMP) family consists of more than 20 secretases or ectocellular enzymes that degrade extracellular matrix proteins, coagulation factors, lipoproteins, latent growth factors, chemokines, and cell adhesion molecules [2] [3]. Dysregulated extracellular matrix (ECM) metabolism is of significance in vascular remodeling during the progression and complication period of atherosclerosis [4]. MMP3 not only degrades ECM components, but also activates MMP1 and other family members [5]. With the advanced progression achieved on DNA technologies, MMP3 gene polymorphisms in the promoter region have been identified. In particular, MMP3 5A/6A is closely related to multiple pathological states [6]. Therefore, we speculated that MMP3 gene polymorphisms may be related to susceptibility of CAD.

Numerous studies have evaluated the involvement of MMP3 gene polymorphisms in CAD; however, the results have often been non-replicable [7] [8] [9]. This may be attributed to racial-specific genetic characteristics, insufficient sample sizes, improper selection of patients and controls, and a lack of adjustments for confounding factors. In this study, we extracted data from eligible literature to assess the involvement of MMP3 -1612 5A/6A in the susceptibility of CAD. Additionally, we explored the heterogeneity among enrolled literature and the existence of publication bias.

## Materials and methods

The meta-analysis of observational research poses a particular challenge due to the inherent biases and differences in research design. Therefore, we performed this analysis in accordance to the guiding principles of the meta-analysis of observational studies in epidemiological statements [10].

### Searching strategy

Relevant literatures on susceptibility of MMP3 - 1612 5A/6A to morbid risk of CAD published before July 2019 were searched in PubMed, Web of Science, Cochrane Library, CNKI, VIP and Wanfang. Key words were searched as follows: MMP3, coronary heart disease/coronary syndrome/myocardial infarction/atherosclerosis and gene/allele/genotype/polymorphism/variation.

### Study selection

Eligible full-text literatures were searched after reviewing titles and abstracts. Citations were manually searched. Data were extracted from recent or most complete publications. When an article contained more than one homogenous features (i.e. the endpoints of CAD and MI), each feature was separately analyzed.

### Inclusion/exclusion criteria

Inclusion criteria included: 1) Independent case-control studies analyzing hospital-based or population-based subjects without any language limitations. 2) Data were complete to obtain or calculate genotype frequencies. 3) Literatures reporting susceptibility of MMP3 -1612 5A/6A to morbid risk of CAD. 4) Genotype distribution in the controls was in accordance to HWE (Hardy-Weinberg equilibrium). 5) None of repeated published data.

Exclusion criteria: 1) Reviews, comments, animal experiments, mechanism researches and case reports. 2) Repeatedly published articles. 3) Studies with inadequate data.

**Figure 1:**
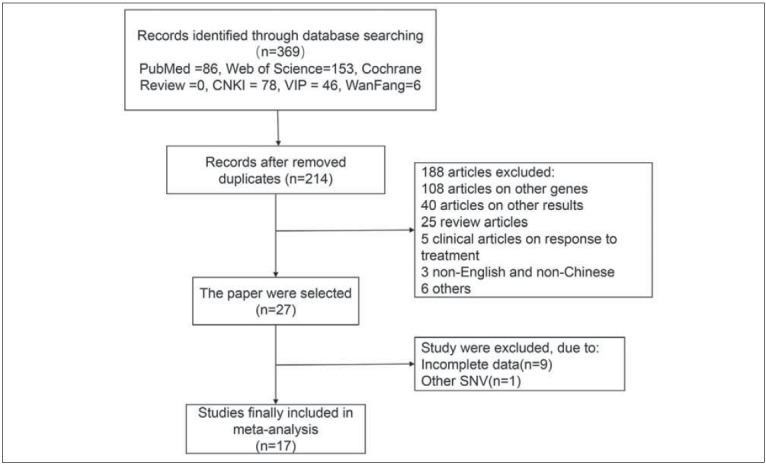
Flow diagram of the publication selection process.

Flow diagram of the publication selection was depicted in Figure 1.

### Data extraction

Data were independently extracted and analyzed by two researchers. Any disagreement was solved by the third researcher. The following data were extracted from each literature: 1) Baseline characteristics of enrolled literature, including published magazine, first author, publish time, etc. 2) Baseline characteristics of subjects, including case number, country of publication, genotype number and distribution, HWE, etc.

### Statistical analysis

Published data were synthesized when three or more studies reporting the same gene polymorphism. Once genotype data were provided, χ^2^ test was applied in the control group.

Heterogeneity test was conducted by calculating OR and the corresponding 95% CI with the *I* ^2^ test and the Q test. The pooled OR in studies lacking the heterogeneity was calculated by the fix-effects model; otherwise, a random-effects model was used. Publication bias was evaluated by depicting funnel plots and quantified by Egger’s test. Data analyses were conducted using RevMan 5.3 and STATA12.0 (London, UK).

## Results

### Baseline characteristics of enrolled literatures

Initially, a total of 369 literatures were searched and 155 replicates were excluded. After reviewingtitles and abstracts, 188 irrelevant literatures (108 literatures reporting other genes, 40 reporting other endpoints, 25 reviews, 5 case reports, 3 published in non-English and non-Chinese language and 6 others) were excluded. At last, 9 literatures lacking complete data and one reporting other SNV variation were excluded. A total of 18 eligible literatures were finally enrolled [7] [8] [11] [12] [13] [14] [15] [16] [17] [18]. Searching procedures were listed in Figure 1.

18 eligible literatures were published from 2003 to 2018, and 10 were published in English [1] [7] [9] [11] [12] [13] [15] [16] [8] [19], and the remaining were published in Chinese language [18] [20] [21] [22] [23] [24] [25]. According to the study endpoints, 4 literatures reported acute coronary syndrome (ACS), 3 reported coronary atherosclerosis (CA), 2 reported CAD, 7 reported myocardial infarction (MI), and one was sub grouped into CAD and MI.

### Research characteristics

Among the 18 eligible literatures, 5 were conducted in Caucasian population [7] [12] [14] [15] [19], 8 were in East Asian population (mainly Chinese population) [17] [18] [20] [21] [22] [23] [24] [25], and 4 were in Middle East population [11] [13] [8] [19]. Patients and controls were all hospital-based population, except for four literatures that did not identify resource of controls. Genotype distribution of controls was in accordance to HWE (PHWE>0.05). Detailed information was listed in Table 1.

**Table 1:** Baseline characteristics of enrolled literatures.

	Author	Year	Country	Journal name/<br>publication<br>origin	Genotyping<br>methods	SNP loci<br>(PHWE)	Sample size	Control	Sample
ACS									
	Andrzej Pawlik<br>et al. [14]	2017	Poles	IUBMB Life	PCR-RFLP	-1612 5A/6A<br>(PHWE=0.26)	197<br>(male=142,<br>female=55)	144<br>(male=103,<br>female=41)	Blood
	He et al. [22]	2004	China	Chin J Emerg<br>Med	PCR-RFLP	-1612 5A/6A<br>(PHWE=0.78)	103<br>(male=73,<br>female=30)	100<br>(male=67,<br>female=33)	
	Xiong et al.<br>[25]	2005	China	Chinese J Clin<br>Rehabilitation	PCR-RFLP	-1612 5A/6A<br>(PHWE=0.78)	103<br>(male=73,<br>female=33)	100<br>(male=67,<br>female=33)	
	Yang et al. [18]	2010	China	J Tongji<br>University	PCR	-1612 5A/6A<br>(PHWE=0.06)	270<br>(male=184,<br>female=86)	258<br>(male=162,<br>female=96)	
CA									
	Tamara Djurić<br>et al. [12]	2008	Serbia	Clin Biochem	PCR	-1612 5A/6A<br>(PHWE=0.19)	265<br>(male=158,<br>female=107)	250<br>(male=142,<br>female=108	Blood

### Overall relevance

**Figure 2:**
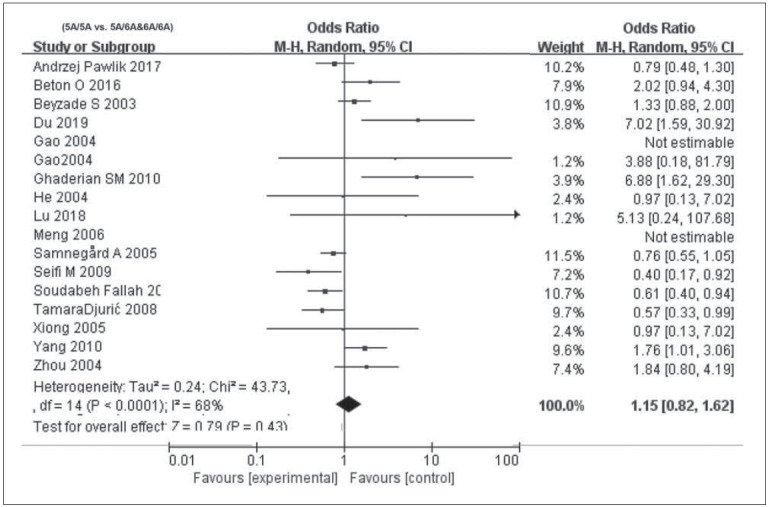
Forest plots demonstrating the association between MMP3 -1612 6A / 5A polymorphism and CAD infection susceptibility in the different models: (5A/5A vs. 5A/6A&6A/6A).

**Figure 3:**
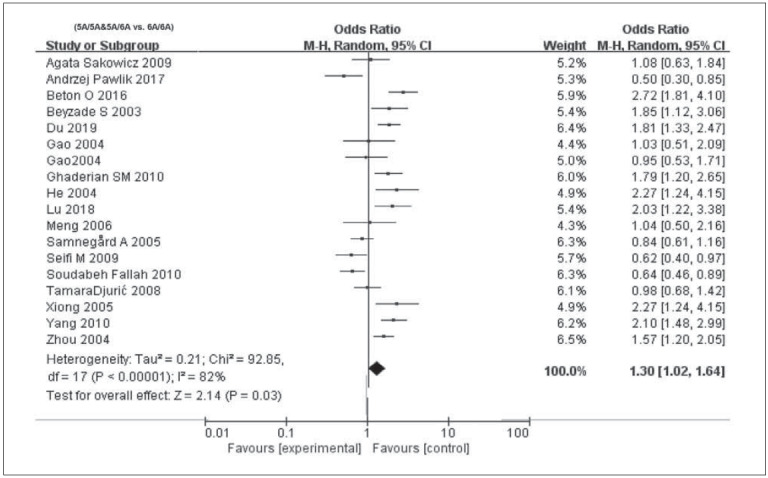
Forest plots demonstrating the association between MMP3 -1612 6A/5A polymorphism and CAD infection susceptibility in the different models: (5A/5A&5A/6A vs. 6A/6A).

**Figure 4:**
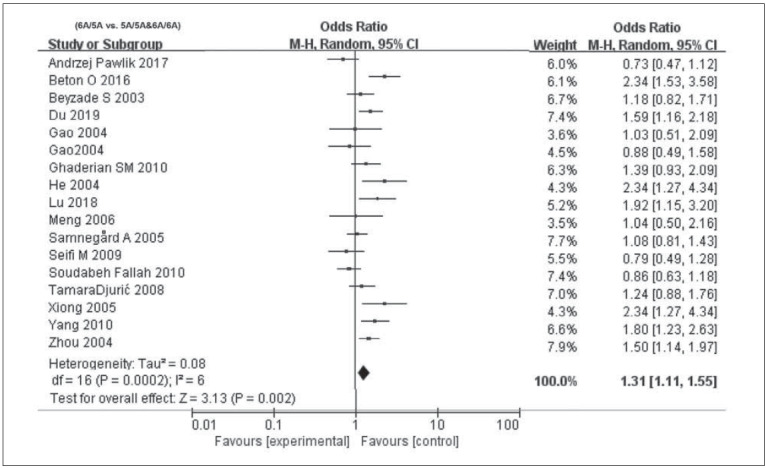
Forest plots demonstrating the association between MMP3 -1612 6A/5A polymorphism and CAD infection susceptibility in the different models: (6A/5A vs. 5A/5A&6A/6A).

Four genetic models were applied for analyzing the involvement of MMP3 -1612 5A/6A in CAD.Heterogeneity existed in every model (*I* ^2^>50%, P<0.05), and thus the random-effects model was used for calculating pooled OR. In recessive model (OR=1.3, 95% CI=1.2–1.64, P=0.03) and overdominant model (OR=1.50, 95% CI=1.14–1.97, P=0.002), MMP3 -1612 5A/6A was correlated to susceptibility of CAD. In the allele model (OR=1.24, 95% CI=1.00–1.54, P=0.05), MMP3 -1612 5A/6A exerted a marginal effect on CAD. After excluding literatures close to HWE violation (P=0.05), no significant changes on overall estimates were observed (Figure 2, Figure 3 and Figure 4). Egger’s test clarified no significant difference in publication bias in the four models (P>0.05, data not shown). To further elucidate the potential factors that may lead to heterogeneity, subgroup analyses were conducted.

### Subgroup analysis based on population

Heterogeneity was absent in four genetic models analyzing the involvement of MMP3 -1612 5A/6A in CAD in East Asian population (*I* ^2^<50%, P>0.05). Mainly in Chinese population, MMP3 -1612 5A/6A gene polymorphism was remarkably linked to CAD (P<0.05, OR>1). Unlikely, no relationship between MMP3 -1612 5A/6A and CAD was observed in Caucasian population and Middle East population (P>0.05). In particular, heterogeneity existed in recessive genetic model (*I* ^2^=77%) and allele model (*I* ^2^=75%) in Caucasian population, as well as four genetic models in Middle East population (*I* ^2^>50%, P<0.05) (Table 2).

**Table 2:** Subgroup analysis based on population.

Regions	Studies<br>(case/controls)	Dominant model		Recessive model		Over-dominant model		Allele model	
		OR<br>(95% CI);<br>P-Value	I^2^<br>(PX^2^)	OR(95% CI);P-Value	I^2^<br>(PX^2^)	OR<br>(95% CI);<br>P-Value	I^2^<br>(PX^2^)	OR<br>(95% CI);<br>P-Value	I^2^<br>(PX^2^)
Caucasian	5<br>(968/1559)	0.84<br>(0.60, 1.17);<br>0.31	58%<br>(0.07)	0.94(0.61, 1.45);0.77	77%<br>(0.005)	1.06<br>(0.87, 1.30);<br>0.57	26%<br>(0.25)	0.92<br>(0.72, 1.19);<br>0.53	75%<br>(0.007)
East Asian	9<br>(1838/1842)	1.94<br>(1.29,2.93);<br>0.002	0%<br>(0.59)	1.68(1.40, 2.02);<0.00001	31%<br>(0.17)	1.57<br>(1.31, 1.88);<br><0.00001	26%<br>(0.22)	1.60<br>(1.39, 1.84);<br><0.00001	14%<br>(0.32)
Middle<br>Eastern	4<br>(1095/913)	1.18<br>(0.44,3.12);<br>0.75	84%<br>(0.0003)	1.31(0.70,2.47);0.40	91%<br>(<0.00001)	1.22<br>(0.76, 1.97);<br>0.42	82%<br>(0.0007)	1.14<br>(0.60, 2.19);<br>0.60	94%<br>(<0.00001)

### Subgroup analysis based on endpoints

MMP3 -1612 5A/6A was significantly linked to CAD under the four genetic models (dominant genetic model: P=0.04/OR=2.13; recessive genetic model: P=0.01/OR=1.91; over-dominant genetic model: P=0.007/OR=0.07; allele model: P= 0.005/OR=1.17) (Table 3). In the meantime, heterogeneity existed in recessive genetic model (*I* ^2^=63%) and allele model (*I* ^2^=52%), and thus a random-effects model was applied. Compared with subjects carrying MMP3 -1612 6A allele homozygous, susceptibility to MI increased 38% in those carrying MMP3 -1612 6A allele heterozygote (1.06–1.80, 0.02), which increased 31% in those carrying MMP3 -1612 5A/6A allele heterozygote (1.11–1.55, 0.0006) (Table 3). A similar association was observed in the allele model of MMP3 -1612 5A/6A. Nevertheless, the individual estimates of risk assessment were heterogeneous, and the possibility of publication bias was also high.

**Table 3:** Subgroup analysis based on endpoints.

Outcomes	Studies<br>(case/controls)	Dominant model		Recessive model		Over-dominant model		Allele	
		OR (95% CI);<br>P-Value	I^2^<br>(PX^2^)	OR (95% CI);<br>P-Value	I^2^<br>(PX^2^)	OR (95% CI);<br>P-Value	I^2^<br>(PX^2^)	OR (95% CI);<br>P-Value	I^2^<br>(PX^2^)
ACS	4<br>(673/602)	1.13<br>(0.67, 1.89);<br>0.64	33%<br>(0.21)	1.52<br>(0.75, 3.11);<br>0.25	87%<br>(<0.0001)	1.60<br>(0.90, 2.82);<br>0.11	81%<br>(0.001)	1.44<br>(0.83, 2.49);<br>0.19	88%<br>(<0.0001)
CA	3<br>(760/763)	0.56<br>(0.41, 0.77);<br>0.0004	0%<br>(0.67)	1.36<br>(0.49, 3.81);<br>0.56	90%<br>(<0.0001)	0.96<br>(0.74, 1.26);<br>0.79	36%<br>(0.21)	0.73<br>(0.59, 0.90);<br>0.004	43%<br>(0.17)
CAD	3<br>(451/494)	2.13<br>(1.02, 4.44);<br>0.04	0%<br>(0.56)	1.91<br>(1.15, 3.16);<br>0.01	63%<br>(0.07)	1.29<br>(1.12, 1.49);<br>0.007	48%<br>(0.15)	1.77<br>(1.19, 2.63);<br>0.005	52%<br>(0.13)
MI	7<br>(2017/2455)	1.87<br>(0.98, 3.56);<br>0.06	76%<br>(0.001)	1.38<br>(1.06, 1.80);<br>0.02	67%<br>(0.006)	1.31<br>(1.11, 1.55);<br>0.0006	10%<br>(0.35)	1.24<br>(1.00, 1.54);<br>0.04	80%<br>(<0.0001)

## Discussion

MMPs are a family of Zn^2+^-dependent neutral proteases that degrade and reshape ECM, thus maintaining their homeostasis [26]. MMP3 has a proteolytic activity on many ECM macromolecules (including different types of collagen) and it is able to promote the transformation of other MMPs. It is reported that MMP1 is upregulated in human atherosclerotic plaques, and ECM degradation activity is also enhanced, suggesting the involvement of MMP1 in CAD and MI [26] [27]. A common deletion/insertion polymorphism (rs3025058) exists in promoter region of human MMP3, which is characterized as 5 or 6 adenosines at 1612 bp upstream of transcription initiation site [28]. Multiple studies have reported that MMP3 rs3025058 is closely linked to coronary atherosclerotic diseases [29] [30] [31].

Previous studies generally believed that MMP3 5A/6A is associated with AS, and the 5A allele is associated with plaque rupture. In this paper, CAD risk in subjects carrying MMP3 -1612 5A homozygous was 30% higher than those carrying MMP3 -1612 6A homozygous. In over-dominant genetic model, MMP3 -1612 5A/6A was a risk factor for CAD. Inconsistent with our findings, a relevant study reported that morbid risk and progression risk are higher in subjects carrying MMP3 -1612 6A homozygous than those carrying MMP3 -1612 5A homozygous [32]. Xu et al. [33] suggested that MMP3 and MMP9 mutations could be utilized as hallmarks for predicting susceptibility to CAD.

Furthermore, subgroup analyses uncovered that MMP3 -1612 6A/5A posed a huge impact on MI or East Asian population, which was consistent with the findings proposed by Want et al. [34] and Niu et al. [4]. Moreover, taken into consideration of CAD as endpoint, MMP3 -1612 6A/5A was significantly related to CAD.

Some limitations in this analysis should be high-lighted. First of all, MMP3 gene polymorphism could be interacted with other known or unknown CAD-associated risk factors. Secondly, we have ruled out many relevant literatures because genotype distribution was failed to be calculated using the inadequate data. Thirdly, sample size was relatively small. Fourthly, additional analyses on baseline characteristics of CAD patients were lacked.

## Conclusions

Collectively, our findings demonstrated that MMP3 -1612 6A /5A was involved in CAD under recessive model and over-dominant model. In East Asian population (mainly Chinese population), MMP3 -1612 6A /5A was susceptibility to CAD in the four genetic models. Our findings required to be validated in a multi-center hospital with larger sample size.

## Dodatak

### Data availability statement

The raw data supporting the conclusions of this article will be made available by the authors, without undue reservation.

### Ethics statement

Not applicable.

### Funding

Not applicable.

### Conflict of interest statement

All the authors declare that they have no conflict of interest in this work.
